# Clinical Heterogeneity of a *TP53* Variant in a Consanguineous Omani Family: A Case Report Featuring a Homozygous Pathogenic Variant

**DOI:** 10.1155/crig/1809799

**Published:** 2026-04-27

**Authors:** Mariya Al Hinai, Chantel Van Wyk, Manal Al Kharusi, Nishath Hamza, Abeer Al Batashi, Abeer Al Sayegh, Maryam Al Shihhi

**Affiliations:** ^1^ National Genetic Centre, Royal Hospital, Muscat, Oman, moh.gov.om; ^2^ Department of Genetics and Genomics, College of Medicine and Health Sciences, United Arab Emirates University, Al Ain, UAE, uaeu.ac.ae; ^3^ Sultan Qaboos Comprehensive Cancer Care and Research Center, University Medical City, Muscat, Oman; ^4^ Clinical Genomics, The Royal Marsden NHS Foundation Trust, Sutton, UK, nhs.uk; ^5^ National Oncology Center, Royal Hospital, Muscat, Oman, moh.gov.om; ^6^ Sheikh Khalifa Medical City, Abu Dhabi, UAE, skmc.ae

**Keywords:** case report, homozygous, Li Fraumeni, *TP53*

## Abstract

**Background:**

Li–Fraumeni syndrome (LFS) is a rare, autosomal dominant cancer predisposition syndrome caused by germline mutations in the *TP53* gene. While heterozygous *TP53* variants are well‐characterized, homozygous germline mutations are extremely rare, and their clinical significance remains poorly understood. Such cases are more likely to arise in consanguineous families, where shared genetic ancestry increases the risk of homozygosity.

**Case Presentation:**

We report a consanguineous Omani family with a homozygous *TP53* missense variant, in a male infant who presented with multiple hypopigmented skin macules and a strong family history of childhood and adult‐onset cancers. Several relatives were identified as heterozygous carriers of the same pathogenic variant. A deceased older sibling exhibited similar cutaneous findings and early malignancy, suspecting he may also have carried the homozygous variant. These skin manifestations may represent a novel phenotypic feature not previously associated with LFS.

**Discussion:**

This case adds to the limited literature on homozygous *TP53* variants and raises the possibility of a link between cutaneous features and homozygosity. While heterozygous carriers often exhibit variable penetrance, the homozygous state may be associated with earlier and more severe phenotypes. Genetic counseling in such families is complex due to uncertainty in predicting clinical outcomes and the psychosocial burden of decision‐making, particularly in children. Challenges in family communication further hinder risk awareness and testing uptake.

**Conclusion:**

This is the first reported case of a homozygous *TP53* p.Arg158His variant in the Omani population, expanding the phenotypic spectrum of LFS. Our findings underscore the importance of genetic counseling, cascade testing, and long‐term surveillance in consanguineous families with hereditary cancer syndromes, and call for further research into genotype–phenotype correlations and associated dermatological findings.

## 1. Introduction

Li–Fraumeni syndrome (LFS, OMIM#151623) is an autosomal dominant inherited cancer syndrome which was first reported by Li and Fraumeni in 1969 and is caused by germline mutations in the *TP53* gene [1, 2]. It is associated with multiple types of tumors in children and adults, including soft tissue sarcoma, osteosarcoma, breast cancer, brain tumors and adrenocortical carcinoma. In 1988, Li et al. [3, 4] described LFS as a proband with a sarcoma aged under 45 years with a first‐degree relative aged under 45 years with any cancer, plus an additional first‐ or second degree relative in the same lineage with any cancer aged under 45 years or a sarcoma at any age. In 1994, Birch et al. [5] identified Li–Fraumeni‐like syndrome (LFS‐L) based on the observation of families that did not fit the classical type of LFS but confirmed the phenotypic heterogeneity of the syndrome. LFS‐L is defined as a proband with any childhood cancer, sarcoma, brain tumor, or adrenocortical carcinoma under the age of 45 years, in addition to a first‐ or second‐degree relative in the same lineage with a typical LFS tumor at any age, as well as an additional first‐ or second‐degree relative in the same lineage with any cancer before the age of 60 years [5]. The lifetime cancer risk associated with LFS is > 70% in males and > 90% in females with a third of tumors present before the age of 20 years [6]. By age 50 years, men with LFS have a 68% risk of developing cancer, with an average age of onset at 40 years. LFS is known for its heterogeneity with variable expressivity and a diverse tumor spectrum, even within the same family. Gargallo et al. mention that the heterogeneity of LFS has been linked not only to the type of *TP53* variant but also to modifier polymorphisms, epigenetic regulation, and genomic instability [7].

### 1.1. Germline *TP53* Variants and LFS

Germline variants in the *TP53* gene have been associated with LFS as described by Malkin et al. in 1990 [2]. *TP53* (OMIM: 191117) is the most frequent somatically altered gene in human cancers [8]. This gene is a tumor suppressor gene and encodes a protein, p53, which is a transcription factor that upregulates the transcription of target genes involved in the cell cycle arrest, DNA repair, apoptosis, and senescence, in response to DNA damage [9]. Currently, *TP53* heterozygous germline variants are the only known cause of LFS, with > 70% of ‘classic’ LFS families identified to have variants in this gene [6]. The IARC *TP53* database identified more than 700 germline variants in the *TP53* gene, and these are expected to increase with advances in next‐generation sequencing (NGS) and as germline genetic testing is more recognized in clinics [10].

Of the variants identified in the *TP53* gene, 74% of germline variants are missense variants, 9% are nonsense variants, and 8% are splice site variants. *TP53* is known to have a hotspot with six of the most common variants found in codons 175, 245, 248, 273, and 282. The majority of variants also occur in the highly conserved DNA‐binding domain [5]. The heterogeneity of LFS has also been linked to certain missense mutations, particularly those within the DNA‐binding domain (e.g., *R175, R248, R273, Y220, R282*), which have been associated with earlier age of tumor onset, dominant‐negative effects, and gain‐of‐function phenotypes that may contribute to more aggressive disease presentations [7].

Germline heterozygous variants in the *TP53* gene are known to cause LFS. While somatic homozygous variants in *TP53* are more commonly observed, homozygous germline variants are very rare. Homozygous *TP53* variants were previously reported in a Brazilian and an Australian article [6, 11]. Brazil is known to have a founder mutation in the *TP53* gene p.Arg337His (c.1010G > A) with a carrier rate of 0.3% in southern and southeastern Brazil, which explains the increased possibility of the occurrence of a homozygous germline *TP53* variant [11]. The Australian article reported a proband from a consanguineous family with a homozygous truncating germline variant in the *TP53* gene Thr18Hisfs ∗ 26 (c.52delA) and a family history consistent with LFS [6].

Details of homozygous missense *TP53* variants have also been reported in patients from the Kingdom of Saudi Arabia. Al Harbi et al. described a homozygous missense *TP53* variant, p.Arg267Trp (c.799C > T), in a 39‐year‐old father of a two‐year‐old boy with a brain tumor [12]. Subsequent genetic testing in the family revealed two additional paternal siblings who were carriers of the homozygous *TP53* variant. Interestingly, all three individuals with the homozygous missense *TP53* variant were phenotypically normal at the time of the study and publication.

Here, we add to the heterogeneity of LFS by reporting a rare homozygous pathogenic germline missense mutation in the *TP53* gene: c.473G > A; p.Arg158His (Exon 5, NM_000546.5) in a consanguineous family from the Sultanate of Oman.

## 2. Case Presentation

**FIGURE 1:**
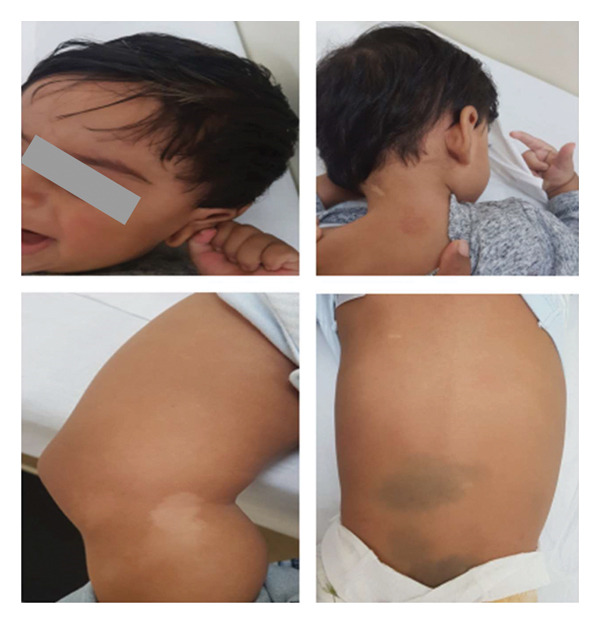
Hypopigmented hair patch, hypopigmented skin macules, and HPO term “dermal melanocytosis.”

The proband, an eight‐month‐old male infant, was referred for clinical genetic evaluation due to the presence of multiple hypopigmented macules with irregular borders (Figure 1), in conjunction with a significant family history of childhood and adulthood cancer. On clinical examination, poliosis, several small to large hypopigmented skin macules, and congenital dermal melanocytosis were observed with posterior neck involvement, abdomen, gluteal region, and lower limbs. No abdominal distension and no other clinically significant features were noted.

**FIGURE 2:**
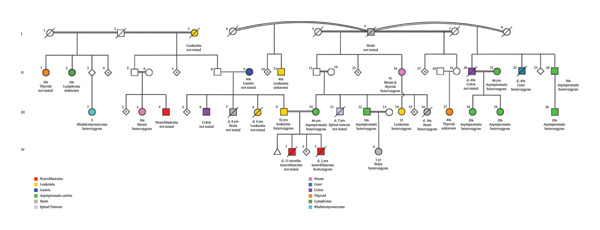
Family pedigree.

This was the couple’s sixth child. They reported an uneventful pregnancy and term birth. The parents (P7G6) had one miscarriage at 9 weeks of gestation and lost a male infant at 11 months of age with neuroblastoma. This infant reportedly had the same hypopigmented skin macules as his brother; however, genetic testing was not requested. Both parents were not reported to have the hypopigmented skin macules. The family history revealed a variety of cancer types, with varying ages of onset among affected relatives as presented in the family pedigree Figure 2 and summarized further in Table 1. This family meets the revised Chompret criteria for a clinical diagnosis of LFS with a history of brain tumors including choroid plexus carcinoma (CPC), colorectal cancer, and liver cancer [9].

**TABLE 1:** Diversity of cancer types and age of onset within family members.

Patient no.	Age of onset	Gender	Type of cancer	Mutation status (homo, hetero, not tested)
I 3	Unknown	Female	Leukemia	Unknown
I 4	Old age	Male	Brain cancer	Not tested
II 1	Adulthood (30s)	Female	Thyroid cancer	Not tested
II 2	Adulthood (30s)	Female	Lymphoma	Unknown
II 9	Adulthood (60+)	Female	Gastric cancer	Not tested
II 14	Adulthood (40s)	Female	Breast cancer (Bilateral) and Thyroid cancer	Heterozygous
II 16	Adulthood (40s)	Male	Leukemia	Unknown
II 19	Adulthood (40s)	Male	Colon cancer	Not tested
II 20	40s	Female	Asymptomatic carrier	Heterozygous
II 21	Adulthood (40s)	Male	Hepatocellular carcinoma	Heterozygous
II 23	50s	Male	Asymptomatic carrier	Heterozygous
III 1	Childhood (5)	Female	Rhabdomyosarcoma	Heterozygous
III 3	Adulthood (30s)	Female	Breast cancer	Heterozygous
III 4	Childhood	Male	Neuroblastoma	Not tested
III 6	Adulthood	Male	Colon cancer	Not tested
III 7	Childhood (8)	Male	Brain cancer	Not tested
III 8	Childhood (6)	Female	Leukemia	Not tested
III 9	Childhood	Male	Leukemia	Heterozygous (father of proband)
III 10	40s	Female	Asymptomatic carrier	Heterozygous (mother of proband)
III 11	Childhood (7)	Male	Spinal tumour	Not tested
III 12	30s	Male	Asymptomatic carrier	Heterozygous
III 14	Adolescent (15)	Female	Leukemia	Heterozygous
III 16	Adulthood (30s)	Female	Brain cancer	Heterozygous
III 17	Adulthood (40s)	Female	Thyroid cancer	Unknown
III 18	20s	Female	Asymptomatic carrier	Heterozygous
III 19	20s	Female	Asymptomatic carrier	Heterozygous
III 20	20s	Male	Asymptomatic carrier	Heterozygous
IV 1	Childhood (11 months)	Male	Neuroblastoma	Not tested
IV 3	Childhood (2) (proband)	Male	Neuroblastoma	Homozygous
IV 5	Childhood (1)	Male	Brain cancer	Heterozygous

The proband was sent for imaging, at 16 months of age, an abdominal ultrasound revealed multiple solid isoechoic lesions in the liver, as well as a solid hyperechoic mass in the right suprarenal region. A computed tomography (CT) scan of the chest, abdomen, and pelvis revealed bilateral adrenal masses, hepatic masses, a right pleural effusion, and small renal lesions. Metaiodobenzylguanidine (MIBG) showed focal avid radiotracer uptake noted in the right adrenal gland and few other foci seen in both lobes of the liver. The left adrenal gland was prominent with no significant MIBG uptake. Histopathological examination and immunophenotyping of a biopsy from the right adrenal mass were consistent with a diagnosis of neuroblastoma. Somatic testing was not done on the tumor. The patient was treated with chemotherapy according to the COG ANB0531 protocol for intermediate‐risk neuroblastoma and underwent tumor resection after the VII cycle of chemotherapy. Unfortunately, 8 months after surgery, the patient experienced a relapse and ultimately succumbed to the disease.

**FIGURE 3:**
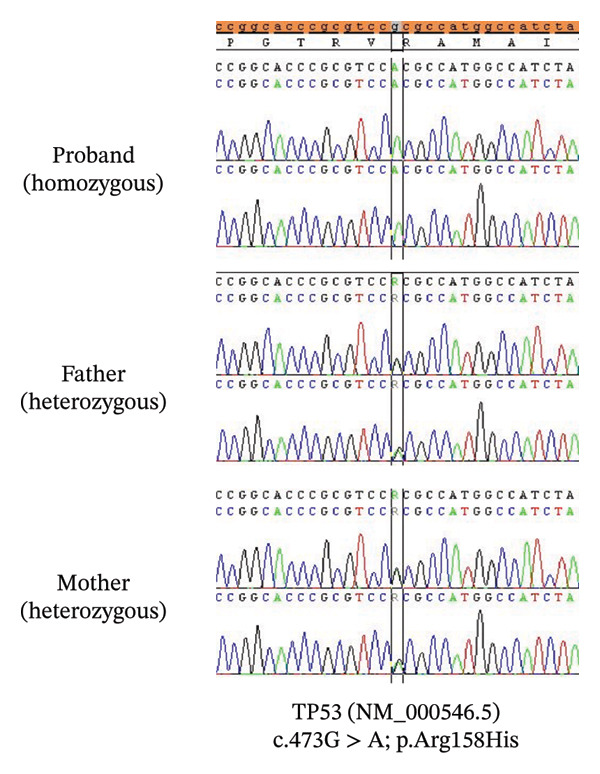
Sequencing chromatograms.

Following confirmation of the cancer diagnosis in the proband, a comprehensive germline cancer panel by NGS was performed and revealed a homozygous pathogenic germline missense variant, NM_000546.6 (*TP53*):c.473G > A (p.Arg158His); chr17:7578457 [hg19] (AF 100% at 399x). This variant has been previously identified as pathogenic in ClinVar and the IARC *TP53* database and is situated within the DNA‐binding domain of *TP53*, a region essential for the protein’s function. According to ACMG/AMP guidelines, the classification considers criteria PS1, PM1, PM2, and PP4, supporting its designation as pathogenic. The homozygous variant in the proband was further confirmed by Sanger sequencing (Figure 3). The parents who are first cousins were confirmed to be heterozygous for the same variant (Figure 3).

The confirmation of a pathogenic variant in the *TP53* gene confirming a molecular diagnosis of LFS with significantly elevated lifetime cancer risk prompted immediate and coordinated action by the genetics team. Recognizing the critical implications of this finding, the team systematically reviewed the family pedigree to identify relatives at potential risk of carrying the same variant. Given the cultural context and the presence of consanguinity within the family, the probability of shared pathogenic variants was elevated. It also made it more likely to identify more individuals with a homozygous genetic profile. At the time of the proband’s diagnosis, only a limited number of cancer cases had been confirmed within the extended family. However, subsequent predictive genetic counseling and testing facilitated the identification of several asymptomatic individuals who were heterozygous carriers of the pathogenic *TP53* variant. Over time, additional family members presented with cancer diagnoses, most commonly leukemia, brain, and thyroid cancer, among others, further supporting the clinical significance and variability of the variant within this pedigree (Figure 2, Table 1).

A continuous review and update of the family pedigree and their health information continue to reveal significant gaps in family history data. The collection of comprehensive medical and genetic information was limited by complex family dynamics and a general reluctance among some relatives to disclose personal health details. In particular, several individuals, especially those from the younger generations, remain unaware of their potential genetic risk and have not yet undergone genetic counseling or testing. These challenges underscore the importance of continued outreach, education, and trust‐building within families affected by hereditary cancer syndromes.

## 3. Discussion

We report an Omani consanguineous family with a homozygous missense *TP53* mutation that could suggest early onset of malignancy in homozygous individuals with cutaneous manifestations reported for the first time.

The germline *TP53* variant identified in this family, c.473G > A (p.Arg158His), is a well‐documented pathogenic mutation. In this case, the proband was found to be homozygous, while several relatives were identified as heterozygous carriers. This variant has been previously reported in association with a spectrum of LFS‐related cancers and has demonstrated segregation with disease in multiple affected families. Notably, previous studies have suggested that this mutation may exhibit reduced penetrance, similar to the *TP53* founder mutation p.Arg337His, potentially accounting for its presence in some clinically unaffected adult carriers within the reported family [11].

While variable penetrance in heterozygous individuals has been well reported, Alharbi et al. (2018) reported a homozygous variant in the *TP53* gene, p.Arg267Trp (c.799C > T), in asymptomatic individuals, one of whom is a father who was 39‐year‐old at the point of publication [12]. He has two sisters, aged 37 and 33 years, who are confirmed homozygous for the variant and are asymptomatic. However, in a heterozygous state, they reported two of the father’s children diagnosed with CPC at a very young age, one at 14 months of age who died at the age of 7 years and the other diagnosed at the age of 2 years (the proband). The father also has two other children who are heterozygous for the variant and were asymptomatic at the point of publication. He has a sister who is confirmed heterozygous and was diagnosed with liver cancer at the age of 49 years. This variability in expression could suggest that other factors mitigate the phenotypic outcome such as environmental factors or additional genetic modifiers, such as telomere attrition or regulatory pathway variants within the p53 regulatory pathway [12]. This differs from our case, where our index patient, who is homozygous for the variant c.473G > A, was diagnosed with neuroblastoma in childhood, and his father, who was heterozygous, was diagnosed with leukemia in his 50s. To date, these findings are not well described in the literature, and further investigations are recommended to understand the variability of age of onset. Our current knowledge makes it difficult to predict the outcome and clinical presentation of homozygous children if both parents carry a *TP53* heterozygous mutation. However, it is recommended that families with confirmed *TP53* pathogenic variants receive comprehensive genetic counseling and follow the *TP53* surveillance [13].

We hypothesize that the unique and similar clinical presentation of our proband to his late deceased brother suggests that the brother may also have carried the same *TP53* missense variant in a homozygous state. To our knowledge, reports of homozygous *TP53* germline mutations remain exceedingly rare. Among these, five cases have been described in association with the Arg337His missense founder mutation, all identified within the Brazilian population. Of these, four developed adrenocortical carcinoma and one CPC, consistent with tumor types commonly associated with LFS. The sixth report is homozygous missense mutations (maternal Arg156His/Arg267Gln; paternal Arg290His), where there was a variable presentation of multiple types of tumors associated with LFS. This further underscores the clinical significance of homozygous *TP53* mutations, although the phenotypic spectrum and penetrance may vary depending on the specific variant. The seventh reported case of a homozygous *TP53* germline mutation involves a truncating variant, associated with a particularly severe clinical phenotype. Notably, that case also documented the presence of multiple pigmented macules on the patient’s lower limbs and back. In comparison, our proband and his deceased brother exhibited multiple hypopigmented patches of skin as well as scalp hair depigmentation, features not commonly described in association with *TP53*‐related syndromes. These observations raise the possibility of a phenotypic correlation between cutaneous manifestations and specific *TP53* variants, particularly in homozygous individuals. Further investigation is warranted to explore the relationship between variant type, severity of malignancy onset, and dermatological findings.

Genetic counseling and testing are particularly critical in consanguineous families, where the risk of inheriting pathogenic variants in the homozygous state is significantly increased due to shared genetic ancestry. In the context of a confirmed *TP53* pathogenic variant, offering both diagnostic and predictive testing to at‐risk relatives becomes essential, not only for individual risk assessment but also for guiding family‐wide cancer surveillance and prevention strategies. This is especially relevant in families with variable age of cancer onset, where early identification of carriers can enable timely clinical interventions. Given the high degree of consanguinity across multiple generations in the reported family, one might have anticipated a greater number of individuals with homozygous *TP53* pathogenic variants, which are associated with more severe phenotypes. These considerations underscore the importance of proactive cascade testing and genetic counseling in consanguineous populations to reduce morbidity and mortality through early detection.

LFS confers a lifelong cancer risk for both children and parents, with variable psychological distress observed within families, complicating decisions about predictive testing. However, family‐based emotional and social support and dyadic coping strategies, including coping in connection, have been associated with reduced stress, improved surveillance adherence, and greater resilience [14, 15]. Further research is needed to better understand family dynamics and psychosocial support needs in families in which both parents carry a heterozygous *TP53* variant.

### 3.1. Limitations

Despite efforts to identify and counsel at‐risk individuals in affected families, gaps in family history remain. It relied on self‐reported cancer histories or limited clinical documentation, and many at‐risk relatives were not tested, which limits segregation analysis and conclusions about penetrance. Additionally, the comprehensive cancer panel for the index patient did not include copy number variant (CNV) analysis, which may have limited variant detection.

## 4. Conclusion

This case study contributes to the limited body of literature on homozygous *TP53* germline mutations by reporting a consanguineous Omani family with a homozygous p.Arg158His variant is associated with early‐onset malignancy and novel cutaneous manifestations. While the pathogenicity of this variant is well‐established in the heterozygous state, its homozygous presentation remains poorly understood, particularly regarding phenotype severity, age of onset, and potential modifiers of disease expression. The dermatological features observed in this case, hypopigmented skin and scalp hair, may represent a previously unreported phenotype in homozygous *TP53* mutation carriers, warranting further investigation.

Given the complexities of genetic risk prediction, especially in consanguineous families where both heterozygous and homozygous carriers may coexist, comprehensive genetic counseling and structured surveillance programs are essential. Moreover, the psychosocial implications of such diagnoses, including variable penetrance, uncertainty in prognosis, and challenges in family communication, underscore the need for a holistic, family‐centered approach. This includes not only medical management but also psychosocial support and educational outreach.

Ongoing research into genotype–phenotype correlations, modifying genetic or environmental factors, and the impact of family dynamics will be critical for improving outcomes in individuals and families affected by LFS. Ultimately, multidisciplinary care integrating genetics, oncology, dermatology, psychology, and social support remains key to the effective management of such complex hereditary cancer syndromes.

## Author Contributions

Mariya Al Hinai: principal author, conceptualized, designed, and drafted the case report’s manuscript. Ensured patient consent and ethical compliance.

Chantel Van Wyk critically revised the manuscript, constructed the pedigree and Table 1, identified and recruited family members, and continually updated the pedigree.

Manal Al Kharusi performed the genetic analysis, interpreted genetic test results, and provided critical revisions related to genetic and molecular aspects and manuscript methodology.

Nishath Hamza performed genetic analysis and interpreted genetic test results.

Maryam Al Shihhi, Abeer Al Sayegh, and Abeer Al Batashi conducted the clinical assessment, collected clinical data and patient history, and reviewed the manuscript.

## Funding

The authors received no financial support for the research, authorship, and/or publication of this article.

## Ethics Statement

Ethical approval was obtained from the Scientific Research Committee at the Royal Hospital, Ministry of Health, Oman. Approval number: MoH/CSR/25/29905.

## Consent

Written consent was obtained from the participants (or their legal guardians) for genetic testing, participation in this research, and the use of clinical images for publication purposes.

## Conflicts of Interest

The authors declare no conflicts of interest.

## Data Availability

The data that support the findings of this study are available on request from the corresponding author. The data are not publicly available due to privacy or ethical restrictions.
